# Optimizing Recovery in Head and Neck Surgery: Factors Influencing Drainage and Early Discharge

**DOI:** 10.3390/jcm15166185

**Published:** 2026-08-10

**Authors:** Ming-Hsun Wen, Tzu-Ang Chen, Tzu-Han Li, Wei-Chen Hung, Ping-Chia Cheng, Chih-Ming Chang, Wu-Chia Lo, Po-Wen Cheng, Po-Hsuan Wu, Li-Jen Liao

**Affiliations:** 1 Department of Otolaryngology, Far Eastern Memorial Hospital, New Taipei City 220216, Taiwanpasteurchen@gmail.com (T.-A.C.); b88401077@ntu.edu.tw (C.-M.C.);; 2 Division of General Medicine, Department of Internal Medicine, Far Eastern Memorial Hospital, New Taipei City 220216, Taiwan; 3 Department of Biomedical Engineering, National Yang-Ming University, Taipei 112304, Taiwan; 4 Department of Electrical Engineering, Yuan Ze University, Taoyuan 320315, Taiwan

**Keywords:** neck surgery, fibrin sealant, postoperative drainage, early discharge, hemostasis

## Abstract

**Background:** Postoperative bleeding and prolonged drainage after head and neck surgery may delay drainage tube removal and hospital discharge, increasing complication risk and resource utilization. Optimizing intra and postoperative hemostasis is essential to facilitate early recovery and support enhanced recovery after surgery (ERAS) pathways. This study aimed to identify factors associated with postoperative drainage and length of hospital stay, with particular focus on energy-based device and fibrin sealant use. **Methods:** Adult patients who underwent neck surgery under general anesthesia with inpatient admission at a tertiary medical center between January 2024 and December 2025 were retrospectively analyzed. Surgical procedures included thyroidectomy (*n* = 99), parotidectomy (*n* = 43), submandibular gland excision (*n* = 25), neck dissection (*n* = 27) and other procedures (*n* = 22). Clinical variables, surgical factors, use of energy-based device, hemostatic agents, postoperative drainage volume, and length of hospital stay were collected. Logistic regression analysis was performed to evaluate predictors of early discharge (defined as discharge on postoperative day 1). **Results:** A total of 216 patients were included. Surgical procedure type was strongly associated with operative duration, postoperative day 1 drainage volume, and length of hospital stay. Neck dissection and total thyroidectomy were associated with increased drainage and prolonged hospitalization. The use of fibrin sealants was independently associated with lower postoperative drainage volume, shorter hospital stay, and a significantly higher likelihood of early discharge (adjusted odds ratio: 4.43, 95% CI 1.92–10.23, *p* < 0.001). Energy-based device use and patient-controlled analgesia were not associated with early discharge. **Conclusions:** Incorporating fibrin sealant into perioperative management may facilitate safer and earlier discharge, improve patient turnover, and optimize resource utilization within ERAS-based care pathways.

## 1. Background

Common head and neck surgeries include thyroidectomy, parotidectomy, submandibular gland excision, and neck lymph node dissection. To ensure patient safety during and after surgery, meticulous control of intraoperative bleeding is essential [1]. Depending on the type of surgery, patient-related factors, and surgeon preference, surgical drains may be placed postoperatively to remove excess fluid and prevent the formation of hematomas or seromas. When the surgeon determines that the drainage volume has decreased sufficiently, the drainage tubes are removed and the patient is discharged. Although surgical drains play an important role in preventing potentially life-threatening complications, shortening the duration of drain retention can reduce drain-related complications (such as wound infection), improve patient comfort, and shorten hospital stay. Prolonged or excessive bleeding may extend the duration of drain placement and increase the length of hospitalization [2,3].

Physical methods that may influence intraoperative blood loss include direct pressure, suturing, ligation, and the use of bone wax. These techniques tamponade bleeding vessels, allowing time for blood coagulation [4]. Thermal techniques use heat to seal blood vessels, typically achieved through electrical current during electrocautery. Chemical methods may involve the use of caustic agents or physiological agents. Caustic agents such as silver nitrate induce hemostasis by denaturing proteins. However, thermal and caustic agents may damage surrounding tissues and impair wound healing. In contrast, surgical tissue adhesives made from biocompatible and biodegradable materials, such as gelatin, cellulose, bovine collagen, thrombin, or fibrin, represent a more physiological approach with fewer complications [5]. Tissue adhesives are effective in controlling mild to moderate bleeding and are often used in combination with other hemostatic techniques, including suturing, ligation, and electrocautery [6,7]. Separately, plant-derived microporous polysaccharide powders act as absorbable topical hemostatic agents that rapidly absorb fluid and concentrate platelets and clotting factors to form a mechanical clot at the bleeding surface, independent of the patient’s coagulation status [8].

In recent years, the concept of enhanced recovery after surgery (ERAS) has gained widespread acceptance [9,10,11]. ERAS integrates perioperative patient care pathways to ensure high-quality, continuous care throughout outpatient diagnosis, pre-admission waiting, preoperative preparation, surgery, postoperative recovery, and post-discharge follow-up. This approach reduces postoperative complications and promotes more efficient utilization of medical resources. Compared with conventional perioperative care, ERAS significantly improves the quality of care before, during, and after surgery. A major advantage of ERAS protocols is that they do not require substantial investment in new hardware; instead, they optimize the use of existing professional manpower and extensively apply evidence-based medical practices without disrupting routine medical services. This significantly enhances anesthetic safety and postoperative recovery quality, making ERAS a concept worthy of promotion in head and neck surgery.

Factors that may affect postoperative blood loss and length of hospital stay include patient age, cardiovascular disease, use of anticoagulants, different surgical procedures, use of energy-based device, application of hemostatic materials (such as Surgicel and fibrin sealant) [12], and postoperative complications. In recent years, shortages of nursing staff and inpatient beds have limited hospital capacity for patient care [13]. Therefore, reducing postoperative bleeding and shortening hospital stays after neck surgery could improve patient turnover and allow more patients to receive inpatient surgical treatment.

Because factors influencing intraoperative and postoperative blood loss and length of hospital stay in neck surgery have not been extensively investigated, this study was proposed with the aim of reducing postoperative bleeding and hospital stay duration, thereby improving patient recovery following neck surgery.

## 2. Materials and Methods

This retrospective cohort study was conducted at a tertiary medical center in accordance with the Declaration of Helsinki and was approved by the Institutional Review Board of Far Eastern Memorial Hospital (approval number FEMH115088-E). Because of the retrospective and anonymized design, the requirement for written informed consent was waived by the same committee. This study enrolled adult patients who underwent neck surgery under general anesthesia with inpatient admission between January 2024 and December 2025. The included surgical procedures comprised thyroidectomy, parotidectomy, submandibular gland excision, lymph node dissection and other neck mass excision.

Data were collected on factors that might have influenced postoperative blood loss and length of hospital stay, including age, type of surgical procedure, surgical duration, intraoperative blood loss, use of energy-based device (LigaSure™ Exact Dissector, Medtronic, Minneapolis, MN, USA), application of fibrin sealant (TISSEEL, Baxter Healthcare Corporation, Deerfield, IL, USA), and use of postoperative patient-controlled analgesia (PCA). Postoperative drainage volume, pain score, time from surgery to drain removal, and length of hospital stay, body-mass index, preoperative coagulation indices (INR, aPTT, and platelet count), and hemoglobin were also recorded. For analysis, patients were grouped by surgical procedure (thyroid lobectomy, total thyroidectomy, parotid extracapsular dissection, superficial parotidectomy, total parotidectomy, submandibular gland excision, neck dissection, and other neck procedures), and the use of fibrin sealant (TISSEEL), energy-based device (LigaSure™ Exact Dissector), and patient-controlled analgesia were each analyzed as dichotomous (use vs. non-use) variables. BMI was categorized as <24, 24–26.9, or ≥27 kg/m^2^ according to Taiwanese reference standards.

In all included patients, hemostasis was achieved intraoperatively before closure. When fibrin sealant was used, the surgical field was thoroughly irrigated and dried before the sealant was applied as a single, thin, uniform layer over the raw surgical bed. Excessive application was avoided in accordance with the manufacturer’s instructions. A 2 mL kit was used for most procedures, whereas a 4 mL kit was selected for larger wound surfaces based on the area requiring coverage. The sealant was allowed to polymerize for approximately 2–3 min before placement of a closed-suction drain (Jackson–Pratt or mini-suction drain). Passive drainage was not used. Drain output was recorded once daily from the first postoperative day (POD) onward, with the POD 1 volume representing output during the first 24 postoperative hours. The drain was removed at the operating surgeon’s discretion—generally when the 24 h output approached or fell below approximately 30 mL, together with the character of the drainage and the patient’s clinical condition—and patients were routinely discharged on the same day; length of hospital stay therefore closely reflected drainage duration, defined as the number of days from surgery to drain removal. Postoperatively, all patients received a standardized maintenance intravenous fluid regimen that was subsequently adjusted according to clinical status.

Patients without inpatient admission, those who did not undergo general anesthesia, patients younger than 20 years of age, and those without surgical drain placement were excluded from the study.

### Statistical Analysis

Descriptive statistical analyses were performed using the Pearson chi-square test to compare categorical variables and the *t*-test to assess statistical differences between continuous variables. Logistic regression analysis was used to evaluate factors associated with the likelihood of early discharge on the first postoperative day. Discharge on the first postoperative day was the sole dichotomous outcome; postoperative drainage volume was analyzed as a continuous variable (in mL). A *p*-value of less than 0.05 was considered statistically significant. For comparisons across the three BMI groups, one-way ANOVA was used. Statistical analyses were performed using Stata/MP version 18 (StataCorp LLC, College Station, TX, USA).

## 3. Results

A total of 216 adult patients who underwent head and neck surgery between January 2024 and December 2025 were included in the analysis and summarized in Table 1. The mean age of the study population was 54.75 ± 13.48 years, with an age range of 23 to 87 years. Of these patients, 113 (52.3%) were older than 55 years, while 103 (47.7%) were younger than 55 years. Female patients constituted a larger proportion of the cohort, accounting for 125 cases (57.87%), whereas 91 patients (42.13%) were male. The mean body-mass index was 25.47 ± 4.83 kg/m^2^, and preoperative coagulation indices were within normal ranges overall (mean INR 0.99 ± 0.05, aPTT 26.83 ± 2.49 s, platelet count 266.13 ± 78.56 × 10^3^/µL, and hemoglobin 13.36 ± 1.81 g/dL). Plasma transfusion and chylothorax did not occur in this cohort, and prior neck irradiation was present in only 2 patients; these factors were therefore not included in the comparative analyses.

Regarding surgical procedures, thyroid lobectomy was the most commonly performed operation, accounting for 81 cases (37.5%). Total thyroidectomy was performed in 18 patients (8.33%). Parotid gland surgeries included extracapsular dissection in 10 patients (4.63%), superficial parotidectomy in 22 patients (10.19%), and total parotidectomy in 11 patients (5.09%). Submandibular gland excision was performed in 25 patients (11.57%), while neck dissection accounted for 27 cases (12.5%). Other types of neck surgeries comprised 22 cases (10.19%).

Energy-based device were frequently used during surgery, with LigaSure™ Exact Dissector applied in 184 patients (85.19%). Hemostatic agents were also commonly utilized; fibrin sealant (TISSEEL) was used in 129 patients (59.72%), whereas 87 patients (40.28%) did not receive this adjunct. Postoperative pain control with PCA was administered in a small proportion of patients, with only 14 cases (6.48%), while the majority (93.52%) did not require PCA.

Postoperative pain levels were generally low, with a mean pain score of 0.45 ± 1.1 on a numeric rating scale ranging from 0 to 10. Most patients (169 cases) reported no pain (pain score = 0), whereas 47 patients experienced some degree of postoperative pain (pain score > 0).

The associations between patient characteristics, surgical factors, and perioperative outcomes, including surgical duration, postoperative day 1 drainage volume, and length of hospital stay, are summarized in Table 2. Overall, the mean surgical duration was 85.69 ± 36.97 min, the mean blood loss was 15.46 ± 13.43 mL, the mean postoperative day 1 drainage volume was 23.13 ± 25.14 mL, and the mean length of hospital stay was 3.12 ± 1.75 days.

Age was not significantly associated with surgical duration, postoperative day 1 drainage volume, or length of hospital stay. Although patients older than 55 years tended to have longer operative times compared with younger patients (90.0 ± 41.08 vs. 80.97 ± 31.39 min), this difference did not reach statistical significance (*p* = 0.073). No significant differences were observed between age groups in postoperative drainage volume or hospital stay duration. Sex was significantly associated with surgical duration and length of hospital stay. Male patients had significantly longer operative times than female patients (94.95 ± 46.32 vs. 78.96 ± 26.54 min, *p* = 0.002). In addition, male patients experienced greater postoperative day 1 drainage volumes (31.07 ± 31.79 vs. 17.35 ± 16.82 mL, *p* < 0.001) and longer hospital stays (3.66 ± 2.19 vs. 2.73 ± 1.21 days, *p* < 0.001). Body-mass index was significantly associated only with surgical duration (*p* = 0.022) and showed no significant association with postoperative day 1 drainage volume or length of hospital stay.

Surgical procedure type was strongly associated with all outcomes (all *p* < 0.001). Neck dissection was associated with the longest surgical duration (135.56 ± 57.27 min), more surgical blood loss (37.04 ± 17.28 mL), highest postoperative day 1 drainage volume (67.27 ± 32.23 mL), and longest hospital stay (6.15 ± 2.43 days). In contrast, less extensive procedures such as parotid extracapsular dissection and submandibular gland excision were associated with shorter operative times, lower drainage volumes, and shorter hospital stays. Thyroid lobectomy demonstrated relatively favorable outcomes compared with total thyroidectomy, which was associated with increased operative duration, drainage volume, and length of hospitalization.

The use of LigaSure™ Exact Dissector was associated with a significantly longer surgical duration compared with cases in which it was not used (87.88 ± 38.67 vs. 73.13 ± 21.47 min, *p* = 0.037). However, LigaSure™ Exact Dissector use did not significantly affect postoperative day 1 drainage volume or length of hospital stay. In contrast, the application of fibrin sealant (TISSEEL) was associated with significantly improved postoperative outcomes. Patients who received TISSEEL had shorter operative times (80.23 ± 28.6 vs. 93.79 ± 45.7 min, *p* = 0.008), lower postoperative drainage volumes (16.89 ± 14.84 vs. 32.38 ± 33.29 mL, *p* < 0.001), and shorter hospital stays (2.69 ± 1.25 vs. 3.76 ± 2.15 days, *p* < 0.001).

Patients who required patient-controlled analgesia (PCA) represented a higher-risk subgroup. PCA use was associated with significantly longer surgical duration (120.0 ± 57.65 vs. 83.32 ± 34.05 min, *p* = 0.0003), substantially higher postoperative day 1 drainage volume (66.14 ± 50.47 vs. 20.15 ± 19.33 mL, *p* < 0.001), and prolonged hospital stay (5.21 ± 3.21 vs. 2.98 ± 1.51 days, *p* < 0.001).

Postoperative pain score was not significantly associated with surgical duration, postoperative drainage volume, or length of hospital stay. Although patients reporting pain scores greater than zero tended to have higher drainage volumes and longer hospital stays, these differences did not reach statistical significance.

Logistic regression analyses were performed to identify selected factors associated with discharge on postoperative day 1 in Table 3. In the univariate analysis, increasing age was associated with a lower likelihood of POD 1 discharge (odds ratio [OR] 0.98, 95% confidence interval [CI] 0.96–1.00, *p* = 0.059); however, this association was no longer statistically significant after adjustment in the multivariate model (OR 0.98, 95% CI 0.95–1.01, *p* = 0.115). Patient sex was not significantly associated with POD 1 discharge in either univariate or multivariate analyses.

Surgical procedure type demonstrated a strong association with POD 1 discharge. Compared with thyroid lobectomy, total thyroidectomy was significantly associated with a reduced likelihood of POD 1 discharge in both univariate (OR 0.11, 95% CI 0.02–0.49, *p* = 0.004) and multivariate analyses (OR 0.09, 95% CI 0.02–0.44, *p* = 0.003). In contrast, parotid extracapsular dissection (ECD) showed a trend toward an increased likelihood of POD 1 discharge in the univariate analysis (OR 7.57, 95% CI 0.92–62.53, *p* = 0.06) and remained a significant independent predictor after adjustment (OR 17.93, 95% CI 1.75–183.82, *p* = 0.015).

Other surgical procedures, including superficial parotidectomy, total parotidectomy, and submandibular gland excision, were not significantly associated with POD 1 discharge in either univariate or multivariate analyses. The “other” surgery category, however, was independently associated with an increased likelihood of POD 1 discharge in the multivariate model (OR 6.54, 95% CI 1.86–23.02, *p* = 0.003), despite not reaching statistical significance in the univariate analysis.

On univariate analysis, body-mass index and the coagulation indices (hemoglobin, aPTT, and platelet count) were not significantly associated with POD 1 discharge and were therefore not entered into the multivariable model. INR showed insufficient variability (0.99 ± 0.05) for meaningful regression and is reported descriptively only.

The use of energy-based device (LigaSure™ Exact Dissector) was not associated with POD 1 discharge in either univariate or multivariate analyses. In contrast, the application of fibrin sealant was strongly associated with an increased likelihood of POD 1 discharge. Patients receiving fibrin sealant were nearly four times more likely to be discharged on POD 1 in the univariate analysis (OR 3.71, 95% CI 2.08–6.62, *p* < 0.001), and this association remained significant after multivariate adjustment (OR 4.43, 95% CI 1.92–10.23, *p* < 0.001).

PCA use was associated with a reduced likelihood of POD 1 discharge in the univariate analysis (OR 0.27, 95% CI 0.07–0.99, *p* = 0.048); however, this association did not persist in the multivariate model (OR 0.89, 95% CI 0.12–6.41, *p* = 0.905), suggesting that PCA use was not an independent predictor after adjustment for other variables.

## 4. Discussion

This study investigated selected factors influencing postoperative bleeding, drainage volume, length of hospital stay, and the likelihood of discharge on postoperative day 1 following common neck surgeries. Our findings demonstrate that surgical procedure type and perioperative hemostatic strategies play a more substantial role in postoperative recovery and early discharge than patient-related factors such as age or sex. These results support the growing emphasis on optimizing intraoperative hemostasis and perioperative care pathways to facilitate enhanced recovery after neck surgery.

Consistent with the existing literature, the extent and complexity of surgery were the strongest determinants of operative duration, postoperative drainage volume, and hospital stay [14]. In our study, neck dissection was associated with significantly longer operative times, more surgical blood loss, higher postoperative day 1 drainage volumes, and prolonged hospitalization, reflecting the extensive tissue dissection and greater lymphatic disruption inherent to this procedure. Similarly, total thyroidectomy resulted in less favorable perioperative outcomes compared with thyroid lobectomy, likely due to the broader surgical field and increased risk of postoperative bleeding. In contrast, less invasive procedures such as parotid extracapsular dissection and submandibular gland excision were associated with lower drainage volumes and shorter hospital stays, highlighting the importance of surgical extent in postoperative recovery.

Although male sex was associated with longer operative times, higher drainage volumes, and longer hospital stays in univariate analyses, sex was not an independent predictor of postoperative day 1 discharge in multivariate models. This suggests that the observed sex-based differences may reflect underlying differences in surgical complexity or case selection rather than sex itself being a direct determinant of recovery. Similarly, increasing age was associated with a reduced likelihood of postoperative day 1 discharge in univariate analysis, but this association did not persist after adjustment, indicating that chronological age alone should not preclude early discharge when other perioperative factors are favorable. In addition, patient-level factors such as body-mass index and preoperative coagulation status were not associated with early discharge, indicating that the benefit of fibrin sealant was independent of these baseline patient characteristics, and that intraoperative hemostasis, rather than patient constitution, was the key modifiable factor influencing early recovery.

One of the most clinically relevant findings of this study is the strong association between the use of fibrin sealant and improved postoperative outcomes. Patients receiving fibrin sealant experienced significantly lower postoperative drainage volumes, shorter operative times, reduced length of hospital stay, and a markedly increased likelihood of discharge on postoperative day 1, even after multivariate adjustment. These findings support the concept that biologically compatible tissue adhesives provide effective hemostasis while minimizing tissue injury, thereby reducing postoperative bleeding and facilitating earlier drain removal [12,15]. In the context of ERAS principles, the routine and judicious use of fibrin sealants may represent a practical strategy to accelerate recovery without requiring additional infrastructure or manpower. This aligns with procedure-specific ERAS evidence for thyroid and parathyroid surgery [16]. We note that comprehensive ERAS protocols for head and neck surgery were developed primarily for major oncological resection with free-flap reconstruction [9]; our findings therefore support the transferable elements applicable to the smaller neck procedures studied here—meticulous hemostasis, minimized drain use with early drain removal, and early discharge—rather than that full pathway. While our findings support earlier drain removal and discharge, sialocele remains a recognized concern in parotid surgery. None was observed in our cohort; as it was not a prespecified endpoint and follow-up was limited, its incidence warrants prospective confirmation.

In contrast, the use of energy-based device such as LigaSure™ Exact Dissector was associated with longer operative duration but did not significantly influence postoperative drainage volume, length of stay, or early discharge. This counterintuitive finding may reflect that surgeons may preferentially select advanced energy devices for inherently more complex or extensive surgeries. Consequently, while LigaSure™ Exact Dissector may improve intraoperative efficiency or safety in complex cases, its use alone is insufficient to reduce postoperative bleeding or facilitate early discharge. These findings underscore that effective postoperative recovery depends not only on surgical instruments but also on adjunctive hemostatic measures and comprehensive perioperative management.

Patients requiring patient-controlled analgesia represented a subgroup with more complex surgical courses, as evidenced by significantly longer operative times, higher postoperative drainage volumes, and prolonged hospitalization. Although PCA use was associated with a reduced likelihood of postoperative day 1 discharge in univariate analysis, this association did not persist after adjustment, suggesting that PCA use is more likely a marker of surgical invasiveness or postoperative burden rather than an independent barrier to early discharge. Notably, postoperative pain scores were generally low across the cohort and were not significantly associated with drainage volume or length of stay, indicating that pain control was effective and unlikely to be a limiting factor for early discharge in this population. Nonetheless, pain was assessed only with a single numeric rating scale, which does not capture analgesic requirements or the temporal pain profile; this limited assessment, together with the uniformly low scores, means pain was not a focus of our analysis.

Taken together, these findings highlight that optimizing intraoperative hemostasis, particularly through the use of fibrin sealants, and selecting appropriate candidates based on surgical extent are key factors in reducing postoperative bleeding and hospital stay after neck surgery. In the context of increasing constraints on nursing manpower and inpatient bed availability, strategies that safely facilitate postoperative day 1 discharge are of considerable clinical and operational value. Future prospective studies are warranted to validate these findings and to integrate standardized hemostatic protocols into ERAS pathways for neck surgery.

Fibrin sealants facilitate hemostasis by mimicking the final steps of the physiological coagulation cascade, forming a stable fibrin clot at the surgical site that effectively seals small blood vessels and lymphatic channels [17]. Unlike thermal or chemical hemostatic methods, fibrin sealants provide localized hemostasis without causing tissue necrosis or excessive inflammatory response, thereby preserving surrounding tissue integrity and promoting wound healing. By reducing oozing from raw surgical surfaces and limiting lymphatic leakage, fibrin sealants can significantly decrease postoperative drainage volume, allowing for earlier drain removal. Consequently, reduced drainage and lower risk of hematoma or seroma formation support earlier mobilization and safe hospital discharge, aligning with ERAS principles and improving overall postoperative care efficiency.

This study has several limitations that should be acknowledged. First, its retrospective, single-center design may introduce selection bias and limit the generalizability of the findings to other institutions with different surgical practices or perioperative care protocols. In addition, as the analysis addressed drainage volume and drain-removal timing, only drained patients were included; since drain placement depends on procedure type and intraoperative findings, the findings may not generalize to non-drained patients. Second, the choice to use hemostatic agents such as TISSEEL or energy-based device was based on surgeon preference rather than randomization, which may confound the observed associations despite multivariate adjustment. Third, heterogeneity in surgical procedures and case complexity, particularly among patients undergoing neck dissection or categorized as “other” surgeries, may have influenced operative duration, postoperative drainage, and length of stay. Additionally, unmeasured factors such as surgeon experience, intraoperative decision-making, postoperative fluid balance, and variations in drain management protocols were not captured and could have affected outcomes. Finally, the relatively small sample size for certain subgroups, including parotid extracapsular dissection and patients receiving PCA, may limit statistical power and widen confidence intervals. From a cost perspective, the direct material cost of TISSEEL should be considered alongside potential savings from earlier discharge, including reduced bed occupancy and nursing workload. Because these costs and savings were not directly measured in the present study and may vary according to local pricing and reimbursement, no conclusion regarding cost-effectiveness can be drawn. Future studies should formally evaluate the incremental cost per early discharge and savings associated with reduced length of stay.

## 5. Conclusions

Our findings highlight that while the extent of the surgical procedure primarily determines postoperative drainage and hospital stay, optimizing intraoperative hemostasis is crucial for facilitating early recovery. Fibrin-sealant use was associated with a higher likelihood of discharge on postoperative day 1 after adjustment for measured covariates. Prospective studies are needed to determine whether this association is causal, safe, and cost-effective.

## Figures and Tables

**Table 1:** Patient Characteristics (Total = 216).

Item	Mean ± SD (%)
Age (years)	54.75 ± 13.48 (23–87)
Sex	
Female	125 (57.87%)
Male	91 (42.13%)
Body Mass Index	25.47 ± 4.83 (15.63–53.71)
<24	88 (40.74%)
24–26.9	64 (29.63%)
≥27	64 (29.63%)
Hemoglobin, g/dL	13.36 ± 1.81 (6.7–17.3)
International normalized ratio	0.99 ± 0.05 (0.88–1.34)
Activated partial thromboplastin time	26.83 ± 2.49 (10.4–33.9)
Platelet, 10^3^/μL	266.13 ± 78.56 (85–915)
Surgery	
Thyroid lobectomy	81 (37.5%)
Total thyroidectomy	18 (8.33%)
Parotid ECD	10 (4.63%)
Parotid Superficial	22 (10.19%)
Parotid total	11 (5.09%)
Submandibular	25 (11.57%)
Neck dissection	27 (12.5%)
Others	22 (10.19%)
Energy-based device (LigaSure™)	
Yes	184 (85.19%)
No	32 (14.81%)
TISSEEL	
Yes	129 (59.72%)
No	87 (40.28%)
Patient-controlled analgesia	
Yes	14 (6.48%)
No	202 (93.52%)
Pain score	0.45 ± 1.1 (0–10)
Postoperative day 1 drainage (mL)	23.13 ± 25.14 (0–160)
Length of hospital stay (days)	3.12 ± 1.75 (1–11)
Day 1 discharge	
No	111 (51.39%)
Yes	105 (48.61%)

**Table 2:** The associations between patient characteristics, surgical factors, and perioperative outcomes.

Item	Surgical Duration (min)	*p*-Value	Surgical Blood Loss (mL)	*p*-Value	Postoperative Day 1 Drainage Volume (mL)	*p*-Value	Length of Hospital Stay (Days)	*p*-Value
Patient factors								
Age (years)	85.69 ± 36.97		15.46 ± 13.43		23.13 ± 25.14		3.12 ± 1.75	
≥55	90 ± 41.08	0.073	16.06 ± 13.64	0.494	21.99 ± 22.06	0.486	3.1 ± 1.63	0.84
<55	80.97 ± 31.39		14.81 ± 13.23		24.38 ± 28.2		3.15 ± 1.88	
Sex								
Female	78.96 ± 26.54	0.002	12.16 ± 9.68	<0.001	17.35 ± 16.82	<0.001	2.73 ± 1.21	<0.001
Male	94.95 ± 46.32		20 ± 16.3		31.07 ± 31.79		3.66 ± 2.19	
Body Mass Index								
<24	90 ± 38.33	0.022	13.92 ± 10.84	0.138	20.15 ± 22.03	0.121	2.99 ± 1.61	0.455
24–26.9	75 ± 26.19		14.84 ± 11.75		21.93 ± 24.81		3.08 ± 1.58	
≥27	90.47 ± 42.26		18.2 ± 17.42		28.43 ± 28.8		3.34 ± 2.07	
Pain-score								
0	84.32 ± 35.7	0.301	15.03 ± 12.46	0.37	21.58 ± 22.46	0.086	3.05 ± 1.64	0.286
>0	90.64 ± 41.25		17.02 ± 16.51		28.7 ± 32.75		3.36 ± 2.1	
Surgical procedure								
Surgery								
Thyroid lobectomy	75.19 ± 21.28	<0.001	10.68 ± 2.93	<0.001	14.64 ± 11.31	<0.001	2.67 ± 1.18	<0.001
Total thyroidectomy	103.33 ± 40.15		22.5 ± 21.44		37.79 ± 33.22		3.33 ± 0.69	
Parotid ECD	81 ± 28.46		9.5 ± 4.38		5.11 ± 2.98		2.1 ± 0.32	
Parotid Superficial	76.36 ± 24.01		12.27 ± 5.28		15.51 ± 10.19		2.59 ± 0.59	
Parotid total	103.64 ± 28.03		20 ± 16.12		25.14 ± 12.1		3.27 ± 1.85	
Submandibular	68.4 ± 20.35		11 ± 6.61		15.89 ± 9.56		2.64 ± 1.08	
Neck dissection	135.56 ± 57.27		37.04 ± 17.28		67.27 ± 32.23		6.15 ± 2.43	
Others	70.91 ± 19.74		9.55 ± 5.75		11.25 ± 11.35		2.36 ± 0.79	
Hemostatic and perioperative factors								
Energy-based device (LigaSure™)								
Yes	87.88 ± 38.67	0.037	15.87 ± 14.04	0.287	23.68 ± 26.29	0.444	3.17 ± 1.84	0.281
No	73.13 ± 21.47		13.13 ± 8.96		19.98 ± 17.13		2.81 ± 1.03	
Fibrin sealant use								
Yes	80.23 ± 28.6	0.008	13.06 ± 10.58	0.001	16.89 ± 14.84	<0.001	2.69 ± 1.25	<0.001
No	93.79 ± 45.7		19.02 ± 16.21		32.38 ± 33.29		3.76 ± 2.15	
PCA								
Yes	120 ± 57.65	<0.001	31.43 ± 25.98	<0.001	66.14 ± 50.47	<0.001	5.21 ± 3.21	<0.001
No	83.32 ± 34.05		14.36 ± 11.41		20.15 ± 19.33		2.98 ± 1.51	

BMI: Body Mass Index; ECD: extracapsular dissection; PCA: Patient-controlled analgesia.

**Table 3:** Logistic regression for early (post OP day 1) discharge.

	Univariate OR (95% CI)	*p*-Value	Multivariate OR (95% CI)	*p*-Value
Patient factors				
Age	0.98 (0.96–1)	0.059	0.98 (0.95–1.01)	0.115
Sex				
Female	1	1	1	1
Male	0.67 (0.39–1.15)	0.150	0.82 (0.39–1.72)	0.607
BMI				
<24	1			
24–26.9	0.81 (0.42–1.54)	0.511		
≥27	0.76 (0.4–1.44)	0.397		
Surgical procedure				
Surgery				
Thyroid lobectomy	1		1	
Total thyroidectomy	0.11 (0.02–0.49)	0.004	0.09 (0.02–0.44)	0.003
Parotid ECD	7.57 (0.92–62.53)	0.060	17.93 (1.75–183.82)	0.015
Parotid Superficial	0.84 (0.33–2.16)	0.719	1.16 (0.4–3.38)	0.780
Parotid total	1.01 (0.28–3.57)	0.989	1.42 (0.33–6.15)	0.641
Submandibular	1.49 (0.59–3.78)	0.395	1.53 (0.57–4.14)	0.399
Neck dissection	N/A *		N/A *	
Others	2.86 (0.96–8.49)	0.059	6.54 (1.86–23.02)	0.003
Hemostatic and perioperative factors				
HGB	0.98 (0.84–1.13)	0.777		
aPTT	1.03 (0.92–1.15)	0.618		
platelet	1.00 (1.00–1.01)	0.321		
Energy-based device (LigaSure™)	0.94 (0.44–1.98)	0.865	1.10 (0.38–3.22)	0.856
Fibrin sealant use	3.71 (2.08–6.62)	<0.001	4.43 (1.92–10.23)	<0.001
PCA	0.27 (0.07–0.99)	0.048	0.89 (0.12–6.41)	0.905

BMI: Body Mass Index; HGB: Hemoglobin; aPTT: activated partial thromboplastin time; ECD: extracapsular dissection; PCA: Patient-controlled analgesia. N/A *: odds ratio not estimable because no POD 1 discharge occurred in the neck dissection group.

## Data Availability

The original contributions presented in this study are included in the article. Further inquiries can be directed to the corresponding author(s).

## Abbreviations

The following abbreviations are used in this manuscript:

BMI	Body Mass Index
HGB	Hemoglobin
INR	International normalized ratio
aPTT	Activated partial thromboplastin time
PCA	Patient-controlled analgesia
ECD	Extracapsular dissection

## References

[1] Muñoz M., Acheson A.G., Bisbe E., Butcher A., Gómez-Ramírez S., Khalafallah A.A., Kehlet H., Kietaibl S., Liumbruno G.M., Meybohm P. (2018). An international consensus statement on the management of postoperative anaemia after major surgical procedures. Anaesthesia.

[2] Harris T., Doolarkhan Z., Fagan J.J. (2011). Timing of removal of neck drains following head and neck surgery. Ear Nose Throat J..

[3] Pennington Z., Lubelski D., Molina C., Westbroek E.M., Ahmed A.K., Sciubba D.M. (2019). Prolonged Post-surgical Drain Retention Increases Risk for Deep Wound Infection After Spine Surgery. World Neurosurg..

[4] Hickman D.A., Pawlowski C.L., Sekhon U.D.S., Marks J., Gupta A.S. (2018). Biomaterials and Advanced Technologies for Hemostatic Management of Bleeding. Adv. Mater..

[5] Shander A., Kaplan L.J., Harris M.T., Gross I., Nagarsheth N.P., Nemeth J., Ozawa S., Riley J.B., Ashton M., Ferraris V.A. (2014). Topical hemostatic therapy in surgery: Bridging the knowledge and practice gap. J. Am. Coll. Surg..

[6] Behrens A.M., Sikorski M.J., Kofinas P. (2014). Hemostatic strategies for traumatic and surgical bleeding. J. Biomed. Mater. Res. Part A.

[7] Spotnitz W.D. (2010). Fibrin sealant: Past, present, and future: A brief review. World J. Surg..

[8] Mariani C., Al Dababsekh A., Carta F., Bontempi M., Barbaccia C., Puxeddu R. (2025). The Use of Microporous Polysaccharide Hemospheres in Thyroid Surgery: A Retrospective Study on Safety and Clinical Outcomes. Medicina.

[9] Nieminen T., Tapiovaara L., Bäck L., Lindford A., Lassus P., Lehtonen L., Mäkitie A., Keski-Säntti H. (2024). Enhanced recovery after surgery (ERAS) protocol improves patient outcomes in free flap surgery for head and neck cancer. Eur. Arch. Otorhinolaryngol..

[10] Ljungqvist O., Scott M., Fearon K.C. (2017). Enhanced Recovery After Surgery: A Review. JAMA Surg..

[11] Małczak P., Pisarska M., Piotr M., Wysocki M., Budzyński A., Pędziwiatr M. (2017). Enhanced Recovery after Bariatric Surgery: Systematic Review and Meta-Analysis. Obes. Surg..

[12] Nguyen M., Tran L., Foreman A., Lockwood C. (2024). The effectiveness of fibrin sealants in head and neck surgery: A systematic review and meta-analysis. Syst. Rev..

[13] Leuchter R.K., Delarmente B.A., Vangala S., Tsugawa Y., Sarkisian C.A. (2025). Health Care Staffing Shortages and Potential National Hospital Bed Shortage. JAMA Netw. Open.

[14] Husted H., Holm G., Jacobsen S. (2008). Predictors of length of stay and patient satisfaction after hip and knee replacement surgery: Fast-track experience in 712 patients. Acta Orthop..

[15] Tabaksert A., James T., Rusius C., Walters H., Lester S. (2024). Drainless, day-case lateral neck dissection with Artiss™ fibrin sealant: A prospective cohort study. Head Neck.

[16] Chorath K., Luu N., Go B.C., Moreira A., Rajasekaran K. (2022). ERAS Protocols for Thyroid and Parathyroid Surgery: A Systematic Review and Meta-analysis. Otolaryngol. Head Neck Surg..

[17] Mankad P.S., Codispoti M. (2001). The role of fibrin sealants in hemostasis. Am. J. Surg..

